# A survey on the practice of phlebotomy in Lithuania and adherence to the EFLM-COLABIOCLI recommendations: continuous training and clear standard operating procedures as tools for better quality

**DOI:** 10.11613/BM.2024.020702

**Published:** 2024-04-15

**Authors:** Ricardas Stonys, Dalius Vitkus

**Affiliations:** 1 Institute of Biomedical Sciences of the Faculty of Medicine, Vilnius University, Vilnius, Lithuania; 2 Centre of Laboratory Medicine of Vilnius University Hospital Santaros Klinikos, Vilnius, Lithuania

**Keywords:** preanalytical phase, phlebotomy, blood specimen collection, healthcare quality assurance

## Abstract

**Introduction:**

The aim of this study was to determine the level of compliance of venous blood sampling (VBS) in Lithuania with the joint recommendations of the European Federation of Clinical Chemistry and Laboratory Medicine and the Latin American Confederation of Clinical Biochemistry (EFLM-COLABIOCLI) and to analyse possible causes of errors. A survey was conducted between April and September 2022.

**Materials and methods:**

A self-designed questionnaire was distributed to the Lithuanian National Societies. Error frequencies and compliance score were computed. Differences between groups were analysed using Pearson’s chi-square, Fisher’s exact criterion, Mann-Whitney U (for two groups), or Kruskal-Wallis (for more than two groups) for categorical and discrete indicators. The association between ordinal and discrete variables was assessed using Spearman’s rank correlation coefficient. Statistical significance was determined at P < 0.05.

**Results:**

A total of 272 respondents completed the questionnaire. Median error rate and compliance score were 31.5% and 13/19, respectively. Significant differences were found among professional titles, standard operating procedures availability, training recency, and tourniquet purpose opinions. A negative correlation was noted between compliance and time since training (r_s_ = - 0.28, P < 0.001).

**Conclusions:**

The findings of this study indicate that there is a significant need for improvement in compliance with the EFLM-COLABIOCLI recommendations on VBS among specialists in Lithuania. Essential measures include prioritizing ongoing phlebotomy training and establishing national guidelines. Harmonisation of blood collection practices across healthcare institutions is crucial.

## Graphical abstract

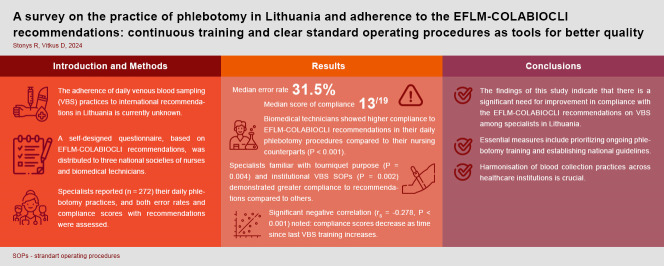

## Highlights

• Venous blood sampling in Lithuania: noncompliance with EFLM-COLABIOCLI

• Inadequate continuous education is linked to poor phlebotomy practices

• Biomedical technicians, trained in phlebotomy, show higher compliance than nurses

• Harmonising blood collection practices across healthcare institutions is crucial

## Introduction

Venous blood sampling (VBS) is the most common invasive procedure performed in healthcare, and the venous blood sample could be considered the most analysed sample in the medical laboratory (*1*). This procedure involves a series of sequential steps that must be executed meticulously to obtain high-quality blood samples for analysis (*1*-*4*).

Errors commonly encountered during the VBS procedure are often associated with patient misidentification, inadequate patient preparation, improper tourniquet application, incorrect order of draw, and sample contamination or inadequate mixing with the additives present in tubes (*1*, *5*-*12*). To minimise the rate of occurrence of these errors, it is crucial to adhere to proper recommendations for phlebotomy. Various guidelines exist for VBS, also recommendations provided by national societies or international organisations in the field of laboratory medicine also guide this practice, including recommendations of the Working Group for Preanalytical Phase (WG-PRE) of the European Federation of Clinical Chemistry and Laboratory Medicine (EFLM) and the Latin American Working Group for Preanalytical Phase (WG-PRE-LATAM) of the Latin America Confederation of Clinical Biochemistry (COLABIOCLI) issued in 2018 (*2*, *4*, *13*, *14*). The EFLM-COLABIOCLI recommendations describe the procedure based on risk and evidence assessment, and grade recommendations for each step based on the quality and strength of the available evidence (*4*). Healthcare professionals in Europe have been encouraged to incorporate these recommendations in their daily practice.

In Lithuania, VBS is mainly performed by nurses and biomedical technicians. However, the training received by these professionals may vary across different educational institutions, as there are no national recommendations or guidelines specifically dedicated to phlebotomy in Lithuania. The absence of such standardised national documents may result in heterogeneity in the phlebotomy procedures carried out by different healthcare professionals in the country.

Current phlebotomy practice in Lithuania and its compliance with international recommendations was unknown prior to this study. Therefore, this study was conducted with the objective of evaluating the level of adherence of VBS procedures to the recommendations set forth by EFLM-COLABIOCLI. The study aimed to identify the most error prone steps, assess the overall quality of phlebotomy procedure, and determine the potential factors contributing to non-compliance with the selected recommendations.

## Materials and methods

### Survey design

For the following study, we utilized a custom-created questionnaire, designed, and revised by laboratory specialists, based on the EFLM-COLABIOCLI recommendations. The questionnaire comprised three main sections. The first section focused on the demographic profile of the participants, including variables such as sex, work experience, education level, and professional title. The second section of the questionnaire covered phlebotomy training, standard operating procedures (SOPs) availability for VBS at respondents’ institutions and other opinion questions. The third section focused on respondents’ routine execution of phlebotomy steps, such as patient and blood tube identification, fasting status verification, sampling, and post-sampling procedures. Examples of the questions (translated into English from Lithuanian) are provided as supplementary material (refer to Supplementary Table 1). The survey was conducted between April and September 2022. The questionnaire was distributed in electronic format *via* Google Forms to the Lithuanian national professional organizations, namely, the Lithuanian Advanced Nursing Practice Association, the Association of Anaesthesia and Intensive Care Nurses, and the Lithuanian Nursing Specialists’ Organization. These organizations are responsible for representing the interests of nurses and biomedical technicians, who are typically involved in phlebotomy procedures in Lithuania. These societies were requested to share the questionnaire with their members. Data were collected anonymously, obviating the need for specific informed consent, and negating the requirement for Ethics Committee approval. It was mandatory to answer all the questions.

### Statistical analysis

All the answers that were not compliant with the recommendations were considered as errors made during VBS on a daily basis and were included into the frequency of errors count. Similarly, less common professional titles (community, mental health, operating room, obstetrician nurses) were grouped as “other professional title nurses”. A total compliance score with EFLM-COLABIOCLI recommendations was calculated for each participant, assigning one point for each compliant response (Q9-Q10 and Q13-Q29), and zero points otherwise. For Q11 and Q12, participants received one point if either question was answered in compliance with recommendations, and zero points otherwise. The maximum achievable compliance score was 19 points. The normality of the compliance score was assessed using the Shapiro-Wilk test, revealing a non-normal distribution. Compliance scores are presented as median and interquartile range (IQR). Differences between groups (education level, professional title, *etc*.) were analyzed using Pearson’s chi-square, Fisher’s exact criterion, Mann-Whitney U (for two groups), or Kruskal-Wallis (for more than two groups) for categorical and discrete indicators. The Kruskal-Wallis *post-hoc* test was employed for pairwise comparisons. The association between ordinal and discrete variables was assessed using Spearman’s rank correlation coefficient. A significance level of P < 0.05 was used to determine statistical significance. The analysis was performed using the Statistical Package for Social Sciences (SPSS Inc, Chicago, IL, USA).

## Results

A total of 272 respondents completed the questionnaire. Most participants were female, consistent with the country’s workforce composition. The basic characteristics of healthcare professionals who perform VBS and participated in the survey are presented in Table 1.

**Table 1:** Basic characteristics of healthcare professionals who participated in the survey

**Variables**	**Number of respondents**
**Sex**	
Male	3 (1.1%)
Female	269 (98.9%)
**Education level**	
Professional bachelor’s degree	187 (68.8%)
Bachelor’s degree	63 (23.2%)
Master’s degree	22 (8.1%)
**Work experience (years)**	
≤ 5	105 (38.6%)
6-15	49 (18%)
≥ 20	118 (43.4%)
**Professional title**	
General practice nurse	201 (73.9%)
Anaesthesia and intensive care nurse	28 (10.3%)
Biomedical technician	21 (7.7%)
Other professional title nurses	22 (8.1%)

Results revealed that majority of respondents had never received training in good phlebotomy practices. Survey findings indicated that the majority reported their institutions had SOPs for VBS, though majority of them misunderstood the purpose of the tourniquet. Table 2 illustrates the distribution of respondents based on these variables.

**Table 2:** Training participation, standard operating procedure awareness, and opinion about tourniquet purpose among healthcare professionals with different titles

**Characteristic**	**Overall** **(N = 272)**	**General practice nurses** **(N = 201)**	**Anaesthesia and intensive care nurse** **(N = 28)**	**Biomedical technician** **(N = 21)**	**Other professional title nurses** **(N = 22)**	**P**
When was the last time you participated in training on venous blood collection?						
≤ 1 year ago	55 (20.2%)	36 (17.9%)	2 (7.2%)	13 (61.9%)	4 (18.2%)	
2*-*4 years ago	61 (22.4%)	47 (23.4%)	6 (21.4%)	3 (14.3%)	5 (22.7%)	0.061*
≥ 5 years ago	53 (19.5%)	35 (17.4%)	9 (32.1%)	3 (14.3%)	6 (27.3%)	
Never attended such training	103 (37.9%)	83 (41.3%)	11 (39.3%)	2 (9.5%)	7 (31.8%)	
Is there a designated standard operating procedure for venous blood collection using a vacuum system in the healthcare facility where you work?						
Available	243 (89.3%)	177 (72.8%)	25 (10.3%)	20 (8.2%)	21 (8.6%)	
Not aware if available or not	23 (8.5%)	19 (82.6%)	2 (8.7%)	1 (4.3%)	1 (4.3%)	0.961*
Not Available	6 (2.2%)	5 (83.3%)	1 (16.7%)	0 (0%)	0 (0%)	
What is the purpose of a tourniquet?						
To highlight veins	115 (42.3%)	79 (39.3%)	12 (57.1%)	14 (66.7%)	10 (45.5%)	
To improve blood flow into the tube	N/A	N/A	N/A	N/A	N/A	0.115^†^
Both options	157 (57.7%)	122 (60.7%)	16 (42.9%)	7 (33.3%)	12 (54.5%)	
*Fisher’s exact test; ^†^Pearson’s Chi-squared test. N/A - no data for the category available.						

The respondents who indicated that they do not verify fasting status prior to VBS or do it occasionally, were asked for the reasons behind such practices. The results showed that the majority of them do not ask about fasting status, believing these tests are unaffected by non-fasting (N = 92; 83%). Others reported lacking knowledge about the importance of fasting (N = 5; 4%) or believed fasting has no impact on any test results (N = 14; 13%).

The frequency of errors is provided in Table 3. The highest error rate was 96.3%, which was observed for the practice of asking the patient to clench or pump their fist. For the question regarding the practice of preparing all the necessary supplies prior to the procedure, only one non-compliant answer was observed, which was likely due to the respondent misunderstanding the question. For this reason, this result was excluded from the median error rate. The observed median error rate for the overall VBS procedure, excluding the aforementioned response, was 31.5%.

**Table 3:** The frequency of errors calculated according to reported phlebotomy practice

**The step of phlebotomy procedure**	**Error frequency, % (N)**
Patient is identified before phlebotomy	2.2 (6)
Patient is identified using open-ended questions	30.1 (82)
Patient’s fasting status is verified prior phlebotomy	41.9 (114)
All necessary supplies are assembled prior to collection	0.4 (1)
Tubes are labelled/identified in the presence of the patient	2.2 (6)
A new, clean pair of gloves is put on before the procedure	8.1 (22)
Tourniquet is placed according to the EFLM-COLABIOCLI recommendations	42.3 (115)
Tourniquet is applied up to 1 minute	30.9 (84)
The patient is not asked to clench or pump their fist	96.3 (262)
If the tourniquet is applied for longer than 1 minute, the phlebotomist releases it, and blood collection is performed on an alternative site	47.8 (130)
The needle is inserted into the vessel at an approximately 5°-30° angle	57.0 (155)
The tourniquet is be removed as soon as the blood starts to flow into the first tube	32 (87)
The order of draw as recommended by the EFLM-COLABIOCLI recommendations is followed	53.3 (145)
Blood is mixed after it has been drawn	13.6 (37)
Filled blood tubes are gently inverted at least 5 times	60.3 (164)
Gentle pressure is applied to the puncture site and the patient is informed to hold it for a period of up to 2 minutes	10.7 (29)
The patient is informed to keep their arm straight	23.2 (63)
The patient is advised to rest for 5 minutes before leaving the phlebotomy unit	34.2 (93)
The correct time of sampling is recorded	13.2 (36)
EFLM-COLABIOCLI - European Federation of Clinical Chemistry and Laboratory Medicine and the Latin American Confederation of Clinical Biochemistry.	

To investigate the potential causes of errors during the phlebotomy procedure, a compliance score analysis was conducted. The median score of compliance with the recommendations was found to be 13 out of 19 (lowest 6, highest 18, interquartile range 4). Participants were categorised into groups based on their level of education, professional titles, work experience, phlebotomy training status, the availability of SOPs in their institutions and opinion on the purpose of tourniquet. The median scores were calculated for each group and their differences were analysed (Table 4). Statistical analysis revealed significant differences among the groups based on professional titles (P < 0.001), the availability of SOP at their institution (P < 0.001), the last time the respondents attended phlebotomy training (P < 0.001) and opinion on the purpose of the tourniquet (P = 0.004). Subsequent pairwise comparisons were conducted to discern significant differences among groups of respondents, and the results are summarized in Table 4.

**Table 4:** The median score of compliance by categories

**Variables**	**Categorical variables**	**Median (IQR) compliance score**	**P**
Last time the specialist had training on good phlebotomy practice	≤ 1 year ago	15 (3)	< 0.001*
	2*-*4 years ago	13 (3)	0.001^‡^
	≥ 5 years ago	14 (3)	< 0.001^§^
	Never attended such training	12 (4)	0.009^║^
Availability of SOP at respondent’s institution	Available	13 (3)	< 0.001*
	Not aware if available or not	12 (3)	0.002^¶^
	Not available	12 (5)	
Education level	Professional Bachelor’s degree	13 (3)	
	Bachelor’s degree	13 (3)	0.286*
	Master’s degree	13 (4)	
Professional title	General practice nurse	13 (4)	< 0.001*
	Anaesthesia and intensive care nurse	13 (4)	< 0.001**
	Biomedical technician	16 (3)	< 0.001^††^
	Other professional title nurses	13 (2)	0.009^‡‡^
Work experience (years)	≤ 5	13 (4)	
	6-15	13 (4)	0.487*
	≥ 20	13 (3)	
Opinion about tourniquet purpose	To highlight veins	14 (3)	
	To improve blood flow into the tube	N/A	0.004^†^
	Both options	13 (4)	
*Kruskal–Wallis H test; ^†^Mann-Whitney U test; Subsequent pairwise comparisons utilized the Kruskal-Wallis H *post-hoc* test. Significant P values, adjusted using Bonferroni correction, are presented for the following group comparisons: ^‡^specialists who attended training ≤ 1 *vs.* 2-4 years ago, ^§^specialists who attended training ≤ 1 year ago *vs.* those who never attended such training, ^║^specialists who attended training ≥ 5 years ago *vs.* those who never attended such training, ^¶^specialists who stated the availability of SOP at their institution *vs.* those who are not aware if it is available or not, **general practice nurses *vs.* biomedical technicians, ^††^anaesthesia and intensive care nurses *vs.* biomedical technicians, and ^‡‡^biomedical technicians *vs.* other professional title nurses. SOP – standard operating procedure. IQR – interquartile range. N/A – no data for the category available.			

In the correlation analysis, a statistically significant negative association was identified between the compliance score and the last time respondent attended training on VBS (r_s_ = - 0.28, P < 0.001). This indicates a noteworthy trend: as the compliance score with recommendations decreases, there is a corresponding increase in time since last training. No other statistically significant associations were found between the compliance score and education level (r_s_ = - 0.07, P = 0.233) or work experience (r_s_ = 0.07, P = 0.249).

## Discussion

To the best of our knowledge, this survey represents the first attempt to evaluate the adherence to international guidelines or recommendations in daily phlebotomy practice in Lithuania.

The findings of this study indicate that the level of compliance with the EFLM-COLABIOCLI recommendations in Lithuania is alarmingly low. The most error-prone steps are the following: patient identification, verification of the patient’s fasting status, tourniquet use and vein selection, the order of draw, and the subsequent mixing of samples. These findings highlight the critical areas where improvements are urgently needed to ensure patient safety and enhance the quality of healthcare services provided. Non-conformity in patient identification could be considered the most critical one. In our investigation, we noted a high incidence of errors in tourniquet use, encompassing incorrect application, delayed release, prolonged application (exceeding 1 minute), and continued blood sampling during prolonged tourniquet use. These inaccuracies in tourniquet application may stem from a misunderstanding of its intended purpose. However, additional studies are warranted to elucidate these assumptions. The findings suggest that ongoing education and standardized SOP for VBS could serve as effective tools for enhancing compliance with international recommendations. This assertion is supported by significant findings showing improved compliance linked to reported practices and variations based on these variables. Furthermore, highlighting preanalytical factors in the education programs for nurses is crucial for understanding their impact on the quality of laboratory results’ and improving current VBS practice compliance with international recommendations. Our study identified that biomedical technicians, being laboratory medicine specialists with practical phlebotomy training in their education, demonstrated higher compliance compared to other nurses’ groups. This may be attributed to the specialized knowledge of biomedical technicians, who are routinely trained by laboratory medicine physicians, medical biologists, and other specialists, emphasizing the awareness of the repercussions of inadequate phlebotomy practices on final test results.

Comparing the results of this study to a previous study conducted by the EFLM WG-PRE on compliance with the CLSI H3-A6 guidelines in 12 European countries, it is concerning to note that the median error rate in Lithuania is even higher than among those countries (*1*). This suggests that compliance of phlebotomy practice with international recommendations in Lithuania is, on average, worse than in other European countries.

Correct patient identification prior to the procedure must be done using open-ended questions as recommended by EFLM-COLABIOCLI (*4*, *6*). While the importance of this step is recognized, various studies suggest that it remains error-prone and is not consistently performed as intended (*1*, *5*, *6*). The discrepancy between current practice in Lithuania and the recommendations raises concerns about the potential of patients being misidentified.

According to recommendations, it is important to check the fasting status of patients before drawing blood samples (*4*). However, the literature indicates that it is not always possible or necessary to collect blood samples in a fasting state depending on the purpose of the test or the level of interference (*15*, *16*). Nevertheless, it is always important to know the fasting status of the patient and interpret the results accordingly. It is also important to note that patients may not be fully aware of the significance of fasting or may not be well informed about the requirement to fast (*7*-*9*). We identified this step as error-prone, and similar error frequencies have been reported in other European countries (*1*).

Tourniquet use is one of the most popular methods for inducing temporary venous stasis, which helps to visualise and select a suitable vein for puncture (*3*). Tourniquet application causes the inner pressure of the vein to increase. It is important to note that prolonged application of a tourniquet can have negative effects, such as tissue hypoxia, pH changes, and alterations in electrolyte balance – particularly potassium levels (*3*, *10*). In our study errors in this step was frequent and these findings align with similar observations in other European countries and are slightly worse than results from Chinese specialists (*1*, *5*). In addition, such findings suggest that the quality of many samples received by Lithuanian laboratories could be compromised, and they could be at a high risk of pseudohyperkalemia, which is of particular concern. It is important to highlight that additional factor, such as strenuous or prolonged fist clenching or pumping, lead to an increase in extracellular potassium due to the depolarisation of skeletal muscle cells (*17*). These factors can have an even greater negative impact when combined with tourniquet application.

Phlebotomists usually must collect multiple tubes of blood during a single venepuncture. While there is some debate regarding the potential for contamination with additives during multi-tube sampling when the order of draw is incorrect, adhering to the recommended order of draw is generally advised (*4*, *18*-*20*). Studies have indicated that contamination is a possibility in such cases and can be challenging to detect (*21*-*23*). Our results reveal that a frequency of errors in this step falls within the range reported by similar studies (8.1-89%), it is noticeably higher than the 8.1% observed in other European countries (*1*, *5*, *12*). Further studies should be carried out to determine the reasons for this difference in error frequency between Lithuania and other European countries. It is plausible that the absence of national guidelines plays a role, as the order of draw may vary among SOPs across different healthcare institutions in Lithuania.

Proper mixing of the additive and blood is essential to ensure accurate test results. According to recommendations, tubes should be inverted at least 5 times to achieve adequate mixing (*4*). Our findings concerning this step is considerably worse than what has been observed in other European countries (*1*). Insufficient mixing, particularly with anticoagulant additives, can elevate the risk of clot formation in samples, resulting in a significant number of clotted samples.

Simply knowing the error rate is insufficient for improving the quality of VBS; it is crucial to identify the sources and reasons behind these errors. Our findings support previous studies that have also demonstrated the association between inadequate training and lower quality of the blood sampling procedure (*24*-*26*). Continuous training is also recommended by the World Health Organisation (WHO) as a best practice for enhancing the quality of blood sample collection (*2*). These findings could be considered as evidence that involving laboratory medicine specialists in VBS training programs could contribute to better-quality procedures. Continuous training could also play a role in harmonising current phlebotomy practices, as it has been shown to improve adherence to guidelines and recommendations (*24*, *25*).

This study acknowledges several limitations that should be taken into consideration when interpreting the findings. Firstly, the questionnaire was designed without undergoing a thorough reliability and validity test. Secondly, the possibility of response bias cannot be disregarded. Participants may have provided answers that were not entirely sincere or accurate, which could affect the reliability of the data. The self-reporting nature of the questionnaire may have influenced participants to provide socially desirable responses or may have led to recall bias. Additionally, the distribution of the questionnaire through electronic means, specifically *via* email, may have excluded a subset of specialists who are less inclined to use information technologies. Also, calculating the response rate is challenging due to uncertainties in the distribution process. The questionnaire was sent to national societies administrations, which then disseminated invitations to regional administrators. The exact number of members to whom the survey was distributed by regional administrators in the regions is unknown, preventing precise determination of the total invited individuals. These limitations could introduce a potential source of selection bias and limit the generalisability of the findings to the entire population of specialists in the country. Nevertheless, questionnaires are a widely used method to assess compliance with various guidelines or recommendations, as well as to gather information on compliance with guidelines for VBS (*1*, *5*, *7*, *9*, *12*, *27*, *28*). Another method that could be used for this purpose is observation studies. This method has the advantage of direct observation of the daily practices of phlebotomists performing VBS and better error determination but is much more difficult to perform on a larger scale or in different regions of the country. Despite these limitations, this study had a relatively large sample size, contributing valuable insights as the first investigation of compliance with the EFLM-COLABIOCLI recommendations on VBS in the country. It is hoped that these findings will stimulate further research and encourage the implementation of more comprehensive studies to advance knowledge and understanding in this area.

In conclusion, the findings of this study indicate that there is a significant need for improvement in compliance with the EFLM-COLABIOCLI recommendations on VBS among specialists in Lithuania. Essential measures include prioritizing ongoing phlebotomy training and establishing national guidelines. Harmonisation of blood collection practices across healthcare institutions is crucial.

## Data Availability

The data generated and analyzed in the presented study are available from the corresponding author upon reasonable request.
